# The Comparative Roles of Mentor Self-Efficacy and Empathy in Fostering Relationship Quality with Youth

**DOI:** 10.1007/s10964-022-01584-7

**Published:** 2022-02-22

**Authors:** Kelsey L. Deane, Ashley A. Boat, Shelley A. Haddock, Kimberly L. Henry, Toni S. Zimmerman, Lindsey M. Weiler

**Affiliations:** 1 Faculty of Education and Social Work, University of Auckland, Auckland, New Zealand; 2 Search Institute, Minneapolis, USA; 3 Department of Human Development and Family Studies, Colorado State University, Fort Collins, USA; 4 Department of Psychology, Colorado State University, Fort Collins, USA; 5 Department of Family Social Science, University of Minnesota Twin Cities, St. Paul, USA

**Keywords:** Youth mentoring, Mentoring self-efficacy, Mentor empathy, Mentoring relationship quality

## Abstract

Youth mentors’ efficacy beliefs and relational skills should both influence the quality of their connections with their mentees, but a lack of research based on large, dyadic and longitudinal samples limits understanding of how mentor characteristics impact relationship quality. This study used three staged and process-focused structural equation models to (1) investigate the mutually reinforcing effects of mentor self-efficacy and empathy over time; (2) compare the longitudinal effects of mid-program mentor efficacy and empathy on end of program mentor and mentee perceptions of relationship quality; and (3) test a similar comparative model using cross-sectional end of program assessments to account for developmental changes in these variables over time. The sample consisted of 664 college-age mentor (76.5% female; ${\bar{x}}$ age = 24.5, range = 21–53; 23.5% non-White) and youth mentee (41% female; ${\bar{x}}$ age = 14.1, range = 10–19; 41.9% non-White) dyads. Mentor empathy predicted mentor perceptions of relationship quality at both time points and mentee perceptions at the end of the program. Mentor efficacy only predicted mentor reported relationship quality at the end of the program. The findings emphasize the importance of investing in empathy training for mentors to support both partners’ positive evaluation of the relationship. Program support to increase mentor self-efficacy should also have added value for mentors.

## Introduction

Because mentors in formal youth mentoring programs commit to supporting younger protégés who often face some kind of disadvantage (Jarjoura et al., 2018), mentors bear the primary responsibility to foster relationship connection (Doty et al., 2019). However, their ability to do this well depends on their motivations, expectations (Strapp et al., 2014), and skills (Spencer et al., 2020). Accordingly, there is a growing need to discern which mentor characteristics are most predictive of relationship quality because these are important mediators of program effectiveness (Rhodes & Lowe, 2009) in a field characterized by small and variable effects (Raposa et al., 2019). Mentoring self-efficacy (Parra et al., 2002, Strapp et al., 2014) and empathy (Spencer et al., 2020) are two mentor characteristics that have repeatedly surfaced in the youth mentoring literature with respect to their influence on mentoring relationship quality. Further, studies have demonstrated that both self-efficacy (Larose, 2013) and empathy (Pryce et al., 2018) are malleable characteristics that can be improved through training. Nevertheless, the existing literature on how mentor characteristics impact relationship quality is limited by a lack of adequately powered longitudinal studies, studies that do not account for both mentor and mentee perspectives of relationship quality, and studies that do not include concurrent measures of mentor self-efficacy and empathy. The field therefore lacks robust findings about the role that each of these characteristics play in facilitating strong mentor–mentee relationships. This study addressed this knowledge gap by examining the comparative effects that mentors’ efficacy beliefs and empathy skills have on their own and their mentees’ perceptions of relationship quality using a large, longitudinal dyadic sample to offer insights for mentor training.

### The Model of Youth Mentoring and the Mentoring-as-Relationship Perspective

A foundational premise of youth mentoring is that a program-mediated relationship between a young person and a more experienced, typically older, person who commits to providing support can contribute to positive developmental outcomes for the mentee (DuBois & Karcher, 2014). Rhodes’ (2005) seminal Model of Youth Mentoring delineates various opportunities through which formal mentoring can produce positive effects for youth participants via cognitive, socioemotional and identity development pathways. Substantial empirical evidence shows mentoring relationships can provide many kinds of youth development opportunities (DuBois et al., 2011). Still, the findings of several meta-analyses of formal mentoring programs have consistently shown that overall program effect sizes for youth outcomes are small (DuBois et al., 2002; DuBois et al., 2011; Raposa et al., 2019). These small effects persist despite a significant growth in mentoring research and more widespread implementation of evidence-informed practices over the past two decades (Raposa et al., 2019).

The limited and variable effectiveness of mentoring programs is more easily understood when considering that each dyadic mentoring relationship within a formal program is itself a relational intervention. Program success is contingent on the collective effectiveness of each dyad. When aggregating effects across many different programs, as is the case for meta-analyses, the overall effect size represents the wide-ranging quality of the hundreds of relationships that constitute the evaluated programs. Floundering relationships obscure the effects of those that are highly impactful. Accordingly, the Model of Youth Mentoring (Rhodes, 2005) purports that high quality relationships, characterized by high trust, mutuality, and empathy, are a necessary precondition for program success. This model is the basis for the mentoring-as-relationship perspective (Cavell & Elledge, 2014).

Although a current contemporary debate within the youth mentoring literature questions the predominant emphasis formal programs place on non-specific relational approaches, compared to targeted, skills-based approaches, there is general agreement that a strong relational bond is a necessary ingredient of mentoring success (Christensen et al., 2020). A range of empirical studies conducted over the past two decades also provide support for the mentoring-as-relationship perspective. In one of the earliest quantitative studies to investigate the links between relationship process variables and outcomes for youth, significant associations were found between mentor-reported relationship closeness and mentor perceptions of the benefits of mentoring for their mentees (DuBois & Neville, 1997). The mentors in this study were involved in a service-learning youth mentoring course and a Big Brothers and Big Sisters (BBBS) of America program. A subsequent study focusing on 50 BBBS mentoring dyads used path analysis to test a process-focused model of mentoring and found that a close bond between mentor and mentee was the most proximal predictor of both mentor and mentee reports of perceived mentee benefits and relationship continuity to the expected one-year mark promoted by the program (Parra et al., 2002). Since then, analyses of data collected as part of a large scale randomized controlled trial (RCT) evaluation of school based BBBS programs have demonstrated that higher mentoring relationship quality was directly associated with improved relationships between youth mentees and their parents and teachers, and indirectly associated with youth self-esteem, prosocial behavior, and academic attitudes via its impact on these other close relationships (Chan et al., 2013). Based on a threshold criterion for relationship closeness, BBBS mentees who reported they were in at least “somewhat close” relationships with their mentors also had better academic outcomes (based on teacher and mentee reports) than those who were not (Bayer et al., 2015).

Outside of BBBS programs, research with mentor and mentees involved in Project Youth Connect found that the quality of the relational connection between dyads predicted a range of positive outcomes for the youth participants, including life skills and positive connections with other adults and school eight months after beginning their mentoring relationships (Zand et al., 2009). A follow-up study, 16 months after the program start, showed the mentor–mentee bond had a sustained and positive association with the mentees’ ability to form friendships with other adults (Thomson & Zand, 2010). Given the pivotal role that relationship quality plays in facilitating mentoring program effects, there is good reason to explore the antecedent drivers of mentoring relationship quality.

### Mentor Characteristics and Mentoring Relationship Quality

Although a range of contextual factors, such as program infrastructure (Keller & DuBois, 2019) and family involvement (Spencer & Basualdo-Delmonico, 2014) have been linked to the quality of mentoring relationships and program effectiveness, the characteristics of the individuals central to the dyadic relationship have the most direct impact on the quality of the connection. Mentees’ individual characteristics and background experiences, particularly their interpersonal history, influence the way they engage in, and perceive, relationships with unfamiliar adults (Williamson et al., 2019); however, there is also evidence that a mentee’s behavior (as the mentoring recipient) also depends on their mentor’s interpersonal history and the nature of their mentoring interactions (Larose et al., 2019). Furthermore, as an intervention strategy that sets out to support young people who experience adversity and disadvantage (Jarjoura et al., 2018), it is primarily the responsibility of mentors to foster quality connections with their young mentees (Doty et al., 2019). Therefore, mentor characteristics that influence relationship quality are of particular interest. Self-efficacy theory (Bandura, 1977) offers potential insights as to why a mentor’s efficacy beliefs and ability to be empathetic both play an important role in forming and sustaining positive relationships with young people.

### Empathy, self-efficacy and mentoring

In the context of youth mentoring, a mentor’s ability to demonstrate empathy to a youth mentee is a core indicator of relational competence (Doty et al., 2019). Empathy involves the ability to connect another’s experience with similar experiences of their own to “feel with” the support recipient (Spencer et al., 2020). Consequently, empathy bridges perspectives in a way that facilitates the expression of care and connection and leaves the support recipient with the sense that the support provider “gets them” (Spencer et al., 2020). Aptly, the Model of Youth Mentoring (Rhodes, 2005) identifies empathy as one salient characteristic of high-quality mentoring bonds and numerous studies provide empirical support for this claim (e.g., Doty et al., 2019; Lester et al., 2018; Munson et al., 2015).

Self-efficacy for mentoring, in contrast, is about a mentor’s *beliefs* in their abilities to successfully perform future-focused mentoring tasks and achieve their mentoring goals. According to self-efficacy theory, when baseline competence levels are equivalent, individuals with higher self-efficacy beliefs tend to outperform those with lower self-efficacy because people’s beliefs in their abilities to succeed fuels their motivation and perseverance, especially when confronted with challenges. This increased effort is associated with higher achievement which further enhances self-efficacy and skill development, thus creating an optimizing self-fulfilling cycle (Bandura, 1997).

Consider the theoretical application to the current study. Taking two mentors who are equally able to demonstrate empathy towards their mentees at the beginning of a program, the mentor with higher self-efficacy for mentoring should feel more motivated to forge a strong connection with their mentee, persevere through challenges in the relationship, further master their relational skills, and feel successful as a result. Mentees who have empathetic and highly efficacious mentors who can deal effectively with issues these mentees bring to the relationship, should also feel a greater sense of trust that they are in safe hands. Mentor self-efficacy and empathy should therefore enhance each other and independently contribute to mentoring relationship quality, from the perspective of both mentors and mentees, over time.

The theoretical proposition that mentor self-efficacy should influence persistence despite relational challenges, and thus relationship quality, has been articulated by a few mentoring researchers (e.g., Karcher et al., 2005; Larose, 2013; Strapp et al., 2014), and a small number of empirical studies explore the influence of mentoring self-efficacy on relationship quality or mentee outcomes. For instance, a small group (*n* = 41) of college-aged mentors working with intermediate-school-aged youth reported increases in self-efficacy over the duration of their mentoring experience. Additionally, higher mentoring self-efficacy at the end of this program was associated with higher levels of relationship satisfaction and greater fulfillment of benefits mentors rated as important to them at the beginning of the program (Strapp et al., 2014). The study that tested the dyadic and process-focused path model mentioned earlier demonstrated that the self-efficacy beliefs of the 50 participating BBBS mentors was a significant antecedent contributor to relationship quality, which had flow-on effects for relationship continuation and perceived mentee benefits (Parra et al., 2002). Mentor self-efficacy was directly linked to youth reports of relationship closeness. In addition, mentors with higher self-efficacy reported fewer relationship obstacles, more contact with their mentees, and greater engagement in program activities, the latter of which was directly linked to mentor reports of relationship closeness. An investigation of the relative influence of distal (e.g., program quality and parental involvement) and proximal factors (e.g., mentor motivation and self-efficacy and mentee risk status) on the relationship quality of cross-age peer mentors and mentees revealed that proximal factors were stronger predictors of relationship quality than distal factors and that mentoring high-risk youth can compromise mentors’ self-efficacy beliefs, which is associated with lower mentor ratings of relationship quality (Karcher et al., 2005). In addition, this same study found that mentors’ self-efficacy at the beginning of the program was positively associated with mentees’ reports that they experienced empathy, praise and attention during the program, and that they mattered to their mentors at the end of the program (Karcher et al., 2005). Similar effects have been found in a large-scale study of school-based BBBS programs. Young people deemed to have greater environmental risk had shorter mentoring relationships compared to youth with low environmental risk—but there was no association between environmental risk and relationship duration for youth who had mentors with high self-efficacy (Raposa et al., 2016).

Unfortunately, no studies, have explored the interrelationships between mentor empathy and self-efficacy, nor their comparative effects on mentoring relationship quality. Deeper insight into how these mentor characteristics influence relationship quality would give useful direction for mentoring training.

## Current Study

This study made use of data collected for a large-scale evaluation of Campus Connections (CC). CC is a university-based, service-learning course in which college students serve as mentors for young people at risk of ongoing life challenges and is described in further detail in the subsequent section. Considering the literature summarized earlier, in this article, mentor empathy is positioned as an indicator of relational competence and mentor self-efficacy and empathy are theorized to act on each other in positive, self-fulfilling ways over the duration of the CC program experience. Effective training and mentoring experience in the early stages of the program should increase both the efficacy beliefs and empathic skills of CC mentors. Higher efficacy beliefs would be expected to feed into greater relationship building and support provision efforts which should influence empathy skill development. Equally, the literature suggests that development of empathy skills as the relationship progresses should feed into efficacy beliefs for future mentoring sessions. Whilst interrelated, mentoring self-efficacy and empathy should each uniquely contribute to mentor and mentee perceptions of relationship quality.

Based on this rationale, the fit of three staged and process-focused models were assessed to examine the effects of mentor self-efficacy and empathy on each other over time, as well as their comparative effects on both mentee and mentor reports of relationship quality at the end of the program. First, a cross-lagged model assessing the reciprocal effects of mentor self-efficacy on mentor empathy (and vice versa) tested the prediction that CC mentor self-efficacy levels at mid-program (Week 6) would significantly and positively predict mentor empathy levels at the end of the program (Week 11), and vice versa (Hypothesis 1), while statistically adjusting for the intercorrelation between the two variables at Week 6 and Week 11, as well as the longitudinal paths between the same repeated measures and other significant covariates (described in Measures later). Second, to bolster claims of causal effects and to reduce the impacts of common method bias, a longitudinal model examined the effects of mentor self-efficacy and mentor empathy mid-program on both mentee and mentor reports of relationship quality at the end of the program to test the prediction that CC mentor self-efficacy and mentor empathy levels at Week 6 would independently predict (positively) mentor and mentee relationship quality at Week 11 (Hypothesis 2), also adjusting for intercorrelations between mentor self-efficacy and empathy, and between mentee and mentor reports of relationship quality and other significant covariates. Last, an alternative, cross-sectional model was also examined to test the predictive effects of mentor self-efficacy and mentor efficacy on mentor and mentee reports of relationship quality, all measured at the end of the program. This alternative model accounted for the fact that mentor self-beliefs and skills and mentoring relationships continue to develop until the end of a program; thus, measures at the end of the program may be a better representation of the predictive effects of these antecedent characteristics on end-of-program relationship quality. Hypothesis 3 therefore also assumed that mentor self-efficacy and empathy at Week 11 would each independently predict (positively) mentor and mentee reports of relationship quality (Week 11), while statistically adjusting for intercorrelations between the efficacy and empathy measures, the mentee and mentor reports of relationship quality and other significant covariates.

## Methods

### The CC Program

CC is a 12-week mentoring program designed specifically to support the resilience and life success of youth who have been exposed to significant adversity. CC was developed, and is operated, by faculty from the Marriage and Family Therapy (MFT) graduate program at Colorado State University (CSU). Other program delivery sites now exist; however, this study focuses on data collected from CC mentors and mentees at CSU. The program takes place on a university campus, where youth are matched with a university student mentor. The mentors are undergraduate students enrolled in a range of majors at CSU and are involved in the program as part of a for-credit service-learning course. The course provides intensive training and ongoing support for mentors focused on developing strong mentor–mentee relationship quality as well as targeted skill development for academic functioning and emotional and behavioral self-regulation (Weiler et al., 2014). Mentors participate in 18 hours of training prior to youth mentees beginning the program. Training includes juvenile court observations and orientation to systems from which youth are referred and content focuses on mentoring skill instruction, role playing, cultural competencies, adolescent development, and awareness of diversity, equity, inclusion and social justice issues. Throughout the 12-week mentoring programs, mentors receive ongoing training and supervision by MFT faculty and postgraduate students.

Youth participants are referred to CC by several community agencies (e.g., Office of District Attorney, Juvenile Probation, Department of Human Services, local school district, and the Center for Family Outreach). Trained professionals complete a referral form that includes contact information and a risk assessment. Upon receipt of the referral, trained CC staff conduct a 1-hour intake appointment with youth and a parent/guardian. Youth are eligible to participate in the program if they: (1) are 11–18 years old; (2) experience at least one risk factor listed as a criterion for participation in their intake assessment (e.g., child has experienced homelessness in the last 5 years, child has been sent to the juvenile hall or had contact with police in the last 12 months, the child does not have any close friends at school or in the neighborhood); and (3) are available to participate in CC during the scheduled after-school hours. Following intake, youth can select their mentors from several mentor profiles to facilitate matches based on shared interests.

When program delivery begins, mentor–mentee pairs spend four hours together one evening per week over the 12 weeks. During this time, youth engage in a variety of activities with their mentors, including a 30-minute walk around campus, 1 hour of individualized tutoring and academic support, dinner, and multiple pro-social activities. In addition to providing training and supervision alongside faculty, graduate students enrolled in a Marriage and Family Therapy academic program provide brief therapeutic sessions to mentees, as needed, throughout CC program delivery to further target the mentees’ mental health and self-regulation needs. Readers are directed to Weiler et al. (2013) for further details about the program.

### Participants

The study sample consisted of 664 adolescents and their mentors from a Western US city. Adolescents were recruited for a RCT evaluation of a specific component within the CC mentoring program. All youth involved in CC as mentees were eligible to participate in the study. Of the 664 mentees in the study sample, 59% were male with a mean age of 14.1 years (*SD* = 1.84). A little over half of youth identified as White (58.1%), 24.5% as Hispanic/Latino, 11.0% as multiracial, 3.2% as African American/Black, 1.7% as American Indian/Alaska Native, 0.5% as Asian American, and 1.1% did not report on race/ethnicity.

Mentors were accepted into the program following an application process and criminal background check. All 664 mentors were undergraduate students; the mean age was 24.5 years (*SD* = 2.68). Mentors were mostly female (83.9%). Most self-identified as White (76.5%), 13.3% as Hispanic/Latino, 2.9% as African American/Black, 1.8% as Asian American, 0.3% as Native Hawaiian/Pacific Islander, 5.0% as multiracial, and 0.3% did not report on race/ethnicity. Some 13.3% of mentors were first-year students, 29.3% were sophomores, 27.9% were juniors, and 29.6% were seniors in college.

### Procedure

The Institutional Review Board at CSU approved the study protocol. Beginning in 2016, youth in CC and their parents/guardians were recruited and informed of the study during their intake meeting with a trained staff member. They were assured participation in the study was voluntary and would have no effect on program standing. Parents and youth received $10 gift cards for each survey as an incentive. Informed assent and consent were obtained from youth participants and one of their parents/guardians.

Student mentors in the CC program were recruited and informed of the study during the first week of class. They, too, were assured that participation in the study was voluntary and would not affect their course enrollment or standing. As an incentive for participation, students received course credit for completing questionnaires, and those who opted not to participate were provided with an alternative assignment. After describing study requirements, informed consent was obtained from college student participants. Survey data were collected via Qualtrics, a web-based survey system, immediately before the start of the intervention, at Weeks 3, 6, and 9, and 11 of the 12-week program for mentors and mentees. Note that measures collected at Weeks 6 and 11 were used for the current study.

### Measures

#### Demographics

Demographic covariates assessed at intake included adolescent age, gender (0 = female; 1 = male), and race/ethnicity. Due to the small sample size, mentee race/ethnicity was recoded as a dichotomous variable (0 = youth of color; 1 = White). Mentor gender (1 = male; 2 = female) and age were also included as covariates.

#### Mentee risk

Mentee environmental and individual risk was assessed at baseline using a subscale from a 32-item risk assessment, which has been used in the mentoring literature (Herrera et al., 2013). Parents/guardians reported on the number of environmental risks (20 items) and individual risks (12 items) youth experienced by indicating either 1 (*yes*) or 0 (*no*). Environmental risk assessed economic adversity (e.g., family has difficulty paying bills), family stress (e.g., family member with drug or alcohol problems), and peer difficulties (e.g., no close friends). Individual risk assessed academic challenges (e.g., failing two or more classes), problem behavior (e.g., bullies others), and mental health concerns (e.g., exhibiting depressive symptoms). Items were summed to create a count of the total number of environmental risks and individual risks that youth experienced; higher scores indicated that youth experienced and/or were exposed to a greater number of risks at baseline.

#### Mentor self-efficacy

Mentors self-reported on their self-efficacy beliefs using an adapted measure from the Personal Efficacy Beliefs Scale (Riggs et al., 1994). Mentor self-efficacy was assessed at Week 6 and Week 11. The instrument included six items. Example items included: “I have confidence in my ability to be a Campus Connections Mentor” and “I have the skills needed to perform my role as a Campus Connections Mentor very well.” Mentors responded using a slider scale that ranged from 0 (*disagree*) to 10 (*agree*). Three items were reversed scored. Scores were calculated by taking the mean; higher values indicated greater perceived self-efficacy. Cronbach’s alpha for Week 6 and Week 11 was 0.73.

#### Mentor empathy

Mentors’ empathy was assessed at Week 6 and Week 11 using a nine-item empathy measure adapted from Long (1990). Example items include, “I am good at understanding my mentee’s problems,” and “Before criticizing my mentee, I try to imagine how I would feel in his/her place.” Mentors responded to items using a slider that ranged from 0 (*disagree*) to 10 (*agree*). One item was reversed. Scores were calculated by taking the mean; higher values indicated greater empathy towards the mentee. Cronbach’s alpha at Week 6 and Week 11 was 0.88.

#### Mentoring relationship quality

Both youth and mentor reports, obtained at Week 11, were used to assess level of perceived mentoring relationship quality. An adaptation of the Mentor Alliance Scale (Cavell et al., 2009) was used to assess youth-report. The scale included 16 items. Sample items included, “I tell my mentor about things that upset me” and “I like spending time with my mentor.” Youth rated each item on a 5-point Likert scale ranging from 1 (*never*) to 5 (*always*), with higher scores indicating greater perceived relationship quality. Cronbach’s alpha was 0.88 at Week 11.

A parallel version of the Mentor Alliance Scale was also used to assess mentor-report of mentoring relationship quality. The scale included 14 items. Example items included, “My mentee talks about things that upset him or her” and “My mentee likes spending time with me.” Mentors rated each item using a 4-point Likert scale ranging from 1 (*never*) to 4 (*always*). Cronbach’s alpha was 0.88 at Week 11.

A high Cronbach alpha does not necessarily indicate a unidimensional scale (Shevlin et al., 2000), thus an exploratory factor analysis (EFA) was conducted to examine the structure of both youth-report and mentor-report of mentoring relationship quality. Based on the EFA, three factors were identified for youth-report (i.e., closeness, disclosure, and conflict) and two factors for mentor-report (i.e., closeness and disclosure). Because the researchers were interested in positive mentor relationship quality and desired a consistent measure across mentor and mentee reports, only youth-reported closeness and disclosure were used as indicators of youth-reported mentoring relationship quality. Items from the EFA were then summed to create four subscales. The youth-reported *disclosure* subscale (*α* = 0.90) included four items (e.g., “I tell my mentor about things that upset me”) and the youth-reported *closeness* subscale (*α* = 0.89) included four items (e.g., “I like spending time with my mentor”). The mentor-reported *disclosure* subscale (*α* = 0.92) included five items (e.g., “My mentee talks about things that upset him or her”) and the mentor-reported *closeness* subscale (*α* = 0.86) included seven items (e.g., “My mentee looks forward to our visits”).

#### Mentoring session

Mentoring sessions took place on four different evenings (Monday, Tuesday, Wednesday, and Thursday). On two of the evenings, mentoring dyads participated in a mentor family condition, where four dyads were grouped together with youth of similar ages for homework and dinner, in comparison to working on their own. To account for potential differences between groups, sessions were controlled for in analyses.

### Data Analytic Plan

Preliminary analysis examined study variable distributions, descriptive statistics (e.g., means, standard deviations and bivariate correlations). An initial confirmatory factor analysis (CFA) was estimated with latent self-efficacy and empathy constructs at Weeks 6 and 11 and latent constructs of youth-reported and mentor-reported mentoring relationship quality at Week 11. In the present analyses, items were parceled (sum of subset of scale items) into three indicators to create latent constructs of mentor self-efficacy and mentor empathy at Weeks 6 and 11. Three parcels were created for each of the four latent factors. In most cases, the item parcels consisted of two individual items. Due to an uneven number of items, some parcels consisted of three individual items. Latent constructs of youth-reported and mentor-reported mentoring relationship quality were created using the subscales identified in the EFA described above (i.e., closeness and disclosure). To test the stability of the self-efficacy and empathy factors at Weeks 6 and 11, model fit of the measurement model, metric invariance (invariance of factor loadings across time), and scalar invariance (latent mean change) were examined across time. To assess metric invariance, the factor loadings for mentor self-efficacy and mentor empathy were constrained to be equal across time (from Week 6 to Week 11). The fit of the metric model was then compared with the fit of the configural (baseline) model using a chi-square difference test. To assess the scalar invariance, the first factor mean (mentor self-efficacy and mentor empathy at Week 6) was constrained to zero and the intercepts were constrained to be equal across time. The fit of the scalar model was then compared with the fit of the metric model.

Following the measurement model, the three structural equation models were estimated. For the first cross-lagged model, mentor self-efficacy and empathy at Week 6 served as predictors of mentor self-efficacy and empathy at Week 11. Cross-lagged paths between mentor’s self-efficacy and mentor’s empathy were also included. For the second structural equation model, mentor self-efficacy and empathy at Week 6 were specified as predictors of youth-reported and mentor-reported mentoring relationship quality at Week 11. The third model was the same as the second except for Week 6 mentor efficacy and empathy being replaced by Week 11 measures. All models controlled for mentee gender, age, race/ethnicity, and individual and environmental risk, as well as mentor age and gender, and mentoring evening session. The following fit indices were used to evaluate model fit: (1) comparative fit index (CFI) greater than 0.95; (2) root mean square error of approximation (RMSEA) below 0.06, and (3) standardized root mean square residual (SRMR), below 0.08 indicate good fit (Hu & Bentler, 1999). All analyses were completed in Mplus 7.35 (Muthén & Muthén, 1998–2015).

Of the 664 adolescents and mentors who participated in the study, 86% had complete data on all study variables; whereas 14% were missing data on at least one study variable. The percentage of missing data on study variables ranged from 0% to 9.8%. To account for missing data, models were estimated using full information maximum likelihood (FIML), which uses all available information from the observed data in the SEM analyses. FIML estimates are computed by maximizing the likelihood of a missing value based on observed values in the data (Schafer & Graham, 2002).

## Results

Means, standard deviations, and inter-correlations for study variables are presented in Table 1. The bivariate correlations demonstrate preliminary support for Hypothesis 1 with significant inter-correlations (medium effect sizes) between the observed mentor self-efficacy and empathy measures at Week 6 and Week 11. Table 1 also shows significant inter-correlations between mentor self-efficacy and the two observed indicators of youth-reported relationship quality (closeness and disclosure) at Week 11 (both small effect sizes), as well as with the parallel, mentor-reported relationship quality measures (medium effect size for closeness and small for disclosure). Similarly, mentor empathy at Week 11 is significantly correlated with the youth-reported relationship quality measures (small effect sizes for both indicators) and the mentor-reported measures (large effect size for closeness and medium for disclosure). These results provide preliminary support for Hypothesis 2 and 3.

**Table 1:** Descriptive statistics and bivariate correlations between study variables

Variable	M (SD)	1	2	3	4	5	6	7	8	9	10	11	12	13	14
1. Mentee gender	0.59 (0.49)														
2. Mentee age	14.13 (1.84)	−0.01													
3. Mentee race/ethnicity	0.59 (0.49)	0.01	0.09*												
4. Mentee individual risk	2.93 (2.16)	0.03	0.24***	0.08											
5. Mentee environmental risk	4.12 (2.85)	0.04	−0.07	−0.12**	0.19***										
6. Mentor gender	1.84 (0.37)	−0.35***	0.07	−0.02	−0.02	0.02									
7. Mentor age	24.49 (2.68)	0.07	0.06	−0.02	0.01	−0.04	−0.11**								
8. Mentor self-efficacy Week 6	7.85 (1.31)	−0.02	−0.04	−0.05	−0.07	−0.05	0.04	0.08*							
9. Mentor self-efficacy Week 11	7.91 (1.38)	−0.03	−0.01	−0.03	−0.06	−0.02	0.07	0.06	0.67***						
10. Mentor empathy Week 6	7.73 (1.34)	−0.12**	−0.06	−0.04	−0.07	−0.05	0.03	0.05	0.40***	0.34***					
11. Mentor empathy Week 11	7.95 (1.33)	−0.11**	−0.04	0.02	−0.06	−0.06	0.05	0.06	0.33***	0.36***	0.67***				
12. Closeness YR	4.59 (0.63)	−0.16***	−0.07	0.04	−0.13**	−0.07	0.04	−0.04	0.11**	0.14**	0.08	0.20***			
13. Disclosure YR	3.92 (1.03)	−0.14**	−0.01	0.08	−0.03	0.01	−0.04	0.02	0.08	0.12**	0.11*	0.18***	0.60***		
14. Closeness MR	4.04 (0.66)	−0.18***	−0.10*	0.03	−0.13**	−0.11*	0.05	0.03	0.29***	0.33***	0.39***	0.57***	0.34***	0.31***	
15. Disclosure MR	3.32 (0.91)	−0.25***	−0.10*	0.02	0.12**	0.01	0.14**	−0.02	0.11**	0.18***	0.34***	0.39***	0.12**	0.27***	0.41***

*Note*. *YR* youth-report, *MR* mentor-report

*p < 0.05, **p < 0.01, ***p < 0.001

A SEM approach that accounts for measurement error and the shared variance between latent constructs enables better assessment of the directionality of effects to ascertain if mentor self-efficacy and empathy have mutually reinforcing influences on each other over time to test Hypothesis 1. In addition, it also allows assessment of the comparative predictive effects of mentor self-efficacy and empathy on both youth and mentor reports of relationship quality, providing a more robust test of the theoretical propositions for Hypothesis 2 and 3.

First, a CFA model was estimated to assess the fit of the latent constructs. Mentor self-efficacy and empathy parcel indicators were all significantly related to the latent constructs to which they were assigned with *r*s ranging from 0.70 to 0.90, *p* < 0.001. The model adequately fit the data, RMSEA = 0.055; CFI = 0.932; SRMR = 0.031. Tests of metric and scalar invariance were also met, indicating that measurements of the mentor self-efficacy and empathy latent constructs were consistent over time.

**Fig. 1:**
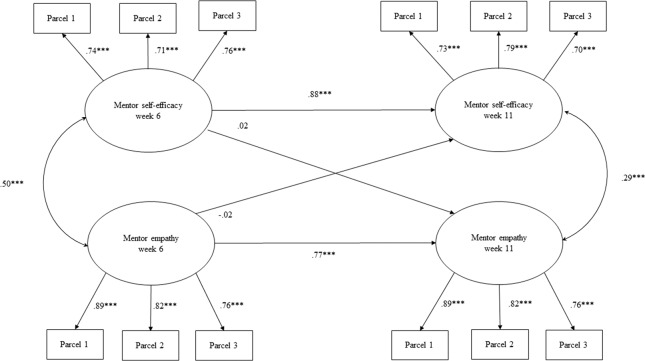
Cross-lagged structural equation model of Week 6 mentor self-efficacy and mentor empathy predicting repeated measures at Week 11. *Note*. Mentee gender, age, individual and environmental and risk were controlled for in the model. Mentor age and gender, as well as evening session were also controlled for in the model. Standardized coefficients are presented. *p < 0.05, **p < 0.01, ***p < 0.001

**Fig. 2:**
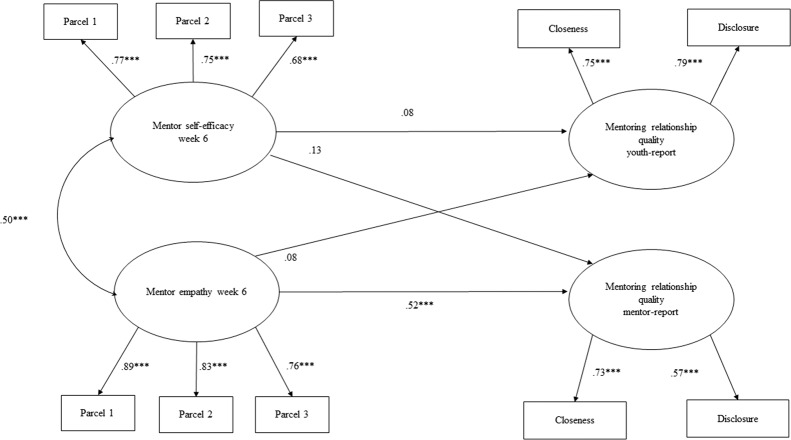
Longitudinal structural equation model of Week 6 mentor self-efficacy and mentor empathy predicting mentor and youth reports of relationship quality at Week 11. *Note*. Mentee gender, age, individual and environmental and risk were controlled for in the model. Mentor age and gender, as well as evening session were also controlled for in the model. Standardized coefficients are presented. *p < 0.05, **p < 0.01, ***p < 0.001

**Fig. 3:**
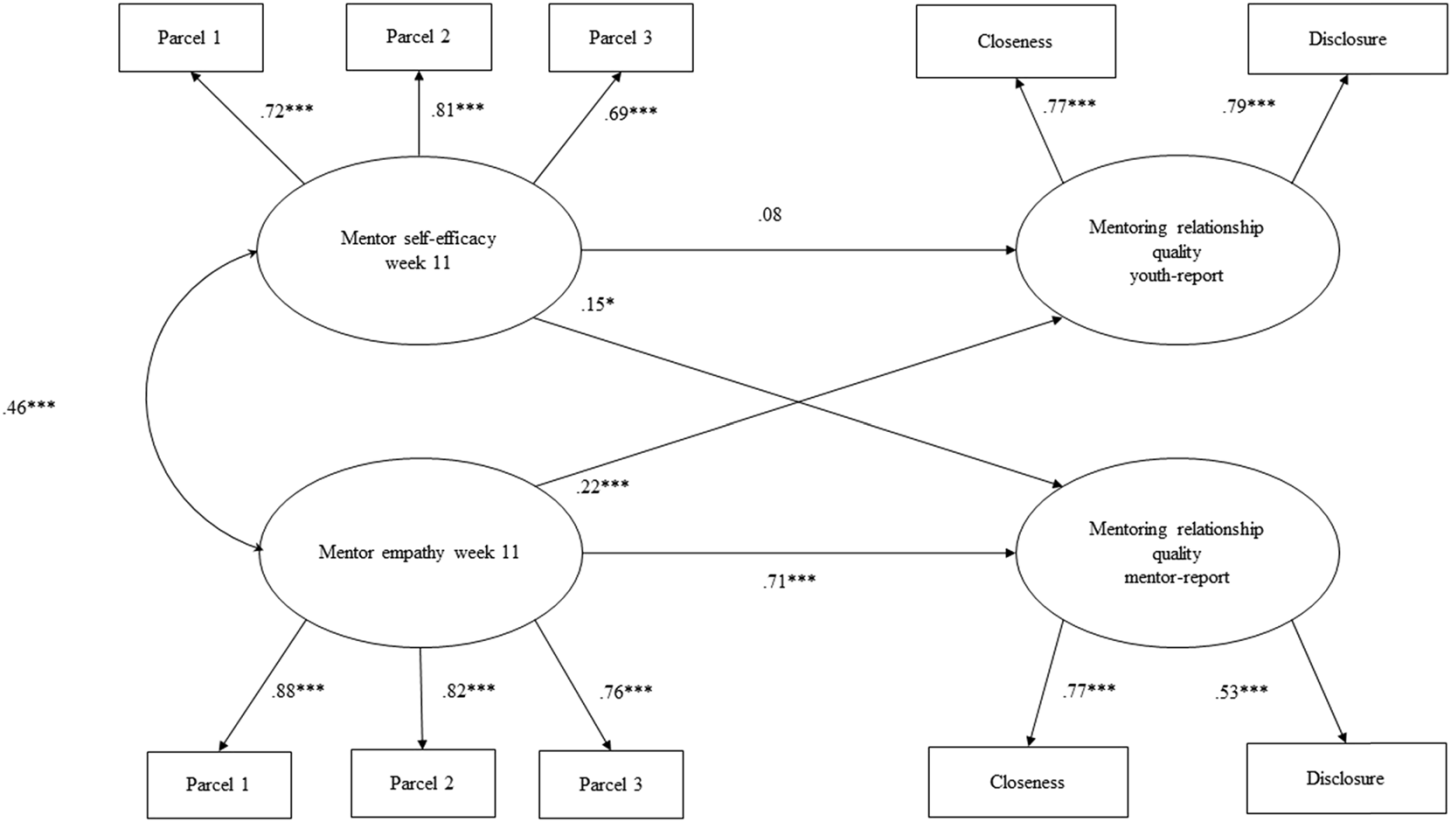
Longitudinal structural equation model of Week 11 mentor self-efficacy and mentor empathy predicting mentor and youth reports of relationship quality at Week 11. *Note*. Mentee gender, age, individual and environmental and risk were controlled for in the model. Mentor age and gender, as well as evening session were also controlled for in the model. Standardized coefficients are presented. *p < 0.05, **p < 0.01, ***p < 0.001

Following the measurement model, the three structural equation models were estimated. The first model demonstrated significant associations between mentor self-efficacy and empathy at each timepoint but nonsignificant cross-lagged effects between Week 6 self-efficacy and Week 11 empathy, as well as between Week 6 empathy and Week 11 self-efficacy (see Fig. 1). The model was an acceptable fit for the data (CFI = 0.931, SRMR = 0.027, RMSEA = 0.059). A second model with good fit (CFI = 0.955, SRMR = 0.029, RMSEA = 0.043) demonstrated that mentor empathy at Week 6 was a significant predictor of mentor-reported relationship quality at Week 11 (*β* = 0.52, *p* < 0.001) but not mentor self-efficacy. Neither mentor self-efficacy nor empathy at Week 6 were significant predictors of youth-reported relationship quality (see Fig. 2). However, the third model demonstrated that higher levels of mentor empathy at Week 11 were significantly associated with higher levels of youth-reported (*β* = 0.22, *p* < 0.01) and mentor-reported (*β* = 0.71, *p* < 0.001) relationship quality. Higher levels of mentor self-efficacy at Week 11 were significantly associated with higher levels of mentor-reported mentoring relationship quality (*β* = 0.15, *p* < 0.01) but not youth-reported mentoring relationship quality at Week 11 (See Fig. 3). This final model was also found to be a good fit for the data (CFI = 0.955, SRMR = 0.030, RMSEA = 0.044). Accordingly, support for Hypothesis 1 and 2 was not confirmed and Hypothesis 3 was partially confirmed.

## Discussion

Despite mentoring self-efficacy and empathy being discussed in the literature as important mentor-driven influences on mentoring relationship quality, no studies have examined their comparative effects to eliminate their potentially confounded influence or their relative influences on mentor and mentee perceptions of their relationship. This study sought to disentangle their comparative effects on both youth and mentor reports of relationship quality using a large, longitudinal sample of matched mentors and mentees from a CC program and concurrent measures of mentor self-efficacy and empathy. The aim was to provide insights to support more effective mentor training and supervision, given previously established links between these mentor characteristics, relationship quality and program outcomes. A staged and process-focused approach first tested the cross-lagged effects of mentor self-efficacy and empathy on each other over time; a second model tested longitudinal predictive effects of mentor efficacy and empathy mid-program on relationship quality at the end of the program; and a final cross-sectional model reassessed the links between mentor efficacy, empathy and relationship quality at the end of the program. Mentor empathy had a more consistent and stronger influence on mentor-mentee relationship quality than self-efficacy; however, mentor empathy was associated with mentoring self-efficacy, and mentors’ self-efficacy beliefs at the end of the program were linked to enhanced mentor perceptions of their relationship at the same time point.

Beginning with the first hypothesis that the two mentor characteristics would have mutually reinforcing longitudinal effects on each other, the findings clearly demonstrate that mentors’ beliefs in their efficacy for mentoring and their perceptions of their empathic skills are interrelated but, for the current sample of mentors, these self-perceptions remained largely stable from the middle to the end of the CC program. Accordingly, after accounting for the shared variance between the Week 6 measures of both latent constructs and the shared variance in the repeated measure of each at Week 11, there was little additional variance to predict over time (as represented by the cross-lagged paths). This is addressed further in the limitations section below.

The findings regarding the comparative effects of mentor self-efficacy and empathy on relationship quality provide some useful insights. Hypothesis 2 focused on testing the longitudinal effects of the mid-program (Week 6) measures of mentor self-efficacy and empathy on end of program (Week 11) perceptions of relationship quality for mentors and mentees because this model addressed the potential temporality confound associated with cross-sectional assessments, and therefore provided a more robust analysis of the directionality of the effects. It also reduced the impact of common method bias whereby significant relationships between measures collected from a participant using the same method (in this case a survey), particularly when administered at the same timepoint, may be artificially inflated rather than a true effect (Jordan & Troth, 2020). The positive predictive effect between Week 6 mentor empathy on mentors’ perceptions of relationship quality at the end of the program provides strong evidence that mentors who feel they are able to demonstrate higher levels of empathy towards their mentee at the mid-way point contributes to their later perceptions of closeness to their mentees and that their mentees can confide in them. Hypothesis 3 assessed an alternative model that, although it did not enable as robust claims about the directionality of the effects, it accounted for the fact that both mentors and mentees and their relationship are in developmental flux as they progress through the program.

Mentor–mentee relationships are acknowledged to move through different stages of development over the course of the relationship and, as mentors and mentees move past initiation of the relationship, they settle into routines and establish communication dynamics that characterize their general pattern of interacting (Keller, 2005). According to social penetration theory, closeness is generated through increasingly intimate self-disclosure and more self-disclosure on the part of a mentor has been found to be associated with higher reports of relationship quality by youth mentees (Dutton et al., 2021), but this can take time. By Week 6 of the Campus Connections program, mentors may feel more comfortable in their roles and begin to draw out more personal disclosures from their mentees that provide more opportunity to be empathetic. Observing deeper engagement from mentees, because of the mentors’ empathic support, likely contributes to mentors’ perceptions that their relationship is increasing in quality. However, mentees may take longer to recognize the increasing quality of the connection that arises from their mentors’ empathic communication. Other research has found that mentor and mentee perceptions of relationship quality are not always well aligned (Dutton et al., 2018). This may explain why there was no significant effect between Week 6 mentor empathy and mentee reports of relationship quality at Week 11. The replicated significant predictive effect of mentor empathy on mentor perceptions of relationship quality with cross-sectional assessment at the end of the program reinforces this important association. In addition, the significant effect between mentor empathy and youth perceptions of relationship quality affirms that, by the end of the program, a mentor’s ability to demonstrate that they can put themselves in their mentees’ shoes to really try to understand them matters to the young person’s willingness to confide in their mentor and feel close to them.

Keeping the focus on Hypothesis 3 and the end-of-program assessments, the mentors’ empathic skills predict both their own and their mentees’ perceptions of relationship quality but mentors’ beliefs in their efficacy for mentoring are only predictive of their own perceptions of relationship quality and not those of their youth mentees. Self-efficacy is an internalized belief that may or may not have outwardly expressed indicators. Therefore, mentors may be effective in their roles from the perspective of an outside observer whilst personally feeling inefficacious. Equally, mentors may feel very efficacious in their role but not be as able to demonstrate specific relational skills in practice. Empathy, on the other hand, involves a support provider being able to both understand and appreciate a support recipient’s perspective and being able to communicate that understanding to them (Hojat, 2009). When comparing both self-efficacy for mentoring and empathic skills, what matters to mentees, in terms of how close they feel to their mentors and how much they are willing to share of themselves, is their mentors’ demonstration that they understand and consider where their mentees are coming from. Mentors’ internal beliefs about their efficacy for mentoring only matter to mentee perceptions insofar as they are, to a degree, linked to mentors’ empathic skills. That stated, the measure of self-efficacy used with CC mentors assesses their beliefs about their ability to be generally effective in their role with CC. The mentor role includes tutoring and academic support, participating and supporting mentees’ engagement in prosocial activities and general positive role modeling, as well as providing more personal emotional support, if needed. Thus, some aspects of the role may not directly engender a sense of closeness and trust for mentees. Once the shared variance between self-efficacy for mentoring and empathy is accounted for, it is empathy that makes a difference for mentees.

This is not to say that efficacy for the other mentoring tasks is not important for other mentee outcomes (e.g., academic and social skills) but, in line with self-efficacy theory, the predictive validity of self-efficacy beliefs for achievement outcomes increases with greater measurement specificity—the specificity of the self-efficacy measure should align with the specific outcome domain of interest (Bandura, 1997). In this study, the interest was in the antecedent drivers of relationship connection because the mentoring-as-relationship hypothesis positions relationship quality as a necessary precondition for other youth outcomes. However, researchers interested in the impact of mentoring self-efficacy beliefs relative to mentoring skills on other outcomes (e.g., mentee academic skills) should use self-efficacy and competency measures specific to the outcome domains of interest (e.g., efficacy for providing academic support).

Despite the broad measure of self-efficacy used in this study, for mentors, the variance component uniquely associated with the self-efficacy construct contributed independently to the mentors’ perceptions of the quality of their relationship (indicated by a sense of closeness and the level of youth disclosure) at the end of the program, as did their perceptions of their empathic skills. When mentors do not feel highly efficacious across all the tasks that constitute their role, they may not see the gains they are making with mentees clearly. The level of connection they feel in their mentoring relationship appears to be dampened compared to those with high efficacy, even if they report being empathetic towards their mentees.

### Limitations

Rather than being a flaw in the proposed theory of self-efficacy in mentoring, the failure to demonstrate evidence of the mutually reinforcing effects of mentor self-efficacy on mentor empathy and vice versa in a longitudinal cross-lagged model likely reflects a problem of model multicollinearity (see Alin, 2010) because there was not substantial change in these measures over the 6–11-week duration between assessment timepoints. This was a key limitation of the current study design. To appropriately test such mutually reinforcing effects, future research would need to capture a sample of mentors where greater change in these self-perceptions were demonstrated over time. As noted above, a more specific measure of self-efficacy for relational engagement would also have enabled a more refined differentiation of the comparative effects of mentors’ self-beliefs vs. their self-reported empathic behavior on both parties’ perceptions of relationship quality and an outside observer’s assessment of mentors’ behavior in this regard would have further reduced potential problems associated with common method bias.

In this study, relationship quality was the outcome of interest because of its positioning in the literature as a critical ingredient for mentoring success. There is, however, growing debate with the field regarding the relative impact of nonspecific relational compared to targeted, evidence-based skill development approaches on young people’s developmental outcomes (Christensen et al., 2020). Future research investigating mentoring efficacy for specific mentoring competencies and comparing the influences of these indicators with empathic skills in both nonspecific and targeted mentoring programs would help to further elucidate the differential effects mentoring self-efficacy and empathy have on program effectiveness.

The cross-sectional assessments of self-efficacy, empathy and relationship quality at Week 11 also limits claims about the directionality of the significant effect between mentor empathy and mentee perceptions of relationship quality. Theoretically, it makes more sense for a mentor’s behavior in the relationship to impact a mentee’s perceptions of the relationship, than for the mentee’s perceptions of relationship quality to influence a mentor to be more empathetic, but the possibility exists for the reverse. An additional assessment of the mentee’s perceptions of relationship quality after program conclusion would help to bolster this claim. Related to this, CC is a time-limited program where mentors and mentees meet each week for four hours but typically only spend a few months together. The differences in the predictive effects at mid-program compared to the end of the program suggest the dynamics between the variables of interest change over the course of this brief, but intensive, program. Many mentoring programs set expectations for a longer match duration, often for a full school or calendar year, and some relationships last much longer. Relationship trajectories in longer matches differ (Pryce & Keller, 2012), therefore it would be worth repeating measures of mentor efficacy, empathy and relationship quality, not only post-program, but over time within longer mentor–mentee relationships to ascertain whether the developmental dynamics of these variables continue to change.

### Implications for Practice

The differential effects that mentor self-efficacy and empathy have on each dyad member’s perspective of relationship quality, as demonstrated in this study, has important implications for mentoring programs. Many programs are constrained by resource limitations (Weinberger, 2014). If there is a need to make decisions about how to invest personnel time, the findings suggest that investment in training and ongoing supervision that focuses explicitly on supporting mentors to effectively demonstrate empathy in their interactions with mentees should be a priority for programs that emphasize the relational connection as a necessary mechanism of change for mentee outcomes. Empathy training should benefit both relationship partners in influencing positive evaluations of relationship quality.

Fortunately, research demonstrates that empathy is teachable (Hojat, 2009, Pryce et al., 2018). Successful training programs using a range of methods, including communication and narrative skills training, role modeling and role playing, and video self-review of professional-client interactions, have been used across different health professions to increase practitioners’ empathic skills (Hojat, 2009). Within the field of mentoring, Facilitating Attuned Interactions (FAN) training (Gilkerson & Pryce, 2020, Pryce et al., 2018) assists mentor support staff to be more attuned in their conversations with mentors. There is substantial overlap between the construct of attunement as conceptualized in the FAN model and the empathic skills training models described by Hojat (2009)—including the ability to read support recipients’ verbal and nonverbal cues, deep listening from an empathic stance, flexibly responding to presenting needs, and self-awareness of one’s own behavioral cues.

The results of the current study also suggest that, when time and resources allow, monitoring mentors’ self-efficacy beliefs and stepping in to support self-efficacy development provides added value for mentors. This is important given the strong theoretical link between self-efficacy beliefs, motivation, and perseverance in the face of adversity (Bandura, 1997). Mentors tasked with supporting young people living in high-risk environments sometimes struggle to sustain enduring relationships but relationship dissolution in such circumstances is less likely when mentors have high self-efficacy (Raposa et al., 2016). Helping mentors develop strong efficacy beliefs may therefore contribute to longer-lasting relationships, an important outcome considering researchers estimate between a third to a half of formal mentoring relationships terminate prematurely and early termination can have detrimental effects for mentees (Spencer et al., 2020).

Like empathy, self-efficacy beliefs are also responsive to intervention efforts, particularly those that provide graduated mastery experiences and role modeling of skills by similar peers (Bandura, 1997). Interestingly, preliminary evidence on the effectiveness of mentoring FAN training indicates it can improve self-efficacy for mentoring along with empathy and attuned communication skills (Pryce et al., 2018). It is promising to see that mentor training and support efforts need not focus on one at the exclusion of the other. This makes sense when considering the inter-relationship between empathic skills and mentoring self-efficacy.

## Conclusion

The Model of Youth Mentoring (Rhodes, 2005) and related empirical research (e.g., Parra et al., 2002) brought into clear focus the strong influence that mentor–mentee relationship quality has on mentoring program success. In dyadic arrangements, both partners to the relationship contribute to the quality of the connection forged and both partners’ perspectives matter to the longevity and impact of the relationship (Spencer, 2007). Nevertheless, mentors have the primary responsibility for making the relationship work for the young person they are supporting. When comparing both mentor self-efficacy and mentor empathy, this study found that empathy is the more powerful of the two when it comes to both mentor and mentee perceptions of relationship quality. Demonstrating empathy is one crucial way that mentors can influence a positive sense of relationship connection for both themselves and their mentees. Importantly, a mentor’s ability to be empathetic towards their mentee is associated with their efficacy beliefs for mentoring, although the directionality of the association is still unclear. Further, high self-efficacy beliefs for mentoring appear to further enhance mentors’ perspectives of relationship quality once the relationship has had sufficient time to develop and the mentor has had time to settle into their role. Capability building that supports strong efficacy for mentoring beliefs should help mentors persevere when faced with challenges and sustain relationships for the benefit of youth mentees. Mentor training and ongoing support efforts that incorporate activities to cultivate strong efficacy beliefs and empathic skills are thus a worthwhile investment.
